# Adaptive behaviour and feedback processing integrate experience and instruction in reinforcement learning

**DOI:** 10.1016/j.neuroimage.2016.08.057

**Published:** 2017-02-01

**Authors:** Anne-Marike Schiffer, Kayla Siletti, Florian Waszak, Nick Yeung

**Affiliations:** a Department of Experimental Psychology, University of Oxford, OX13UD Oxford, UK; b Université Paris Descartes, Sorbonne Paris Cité, Paris, France; c CNRS (Laboratoire Psychologie de la Perception, UMR 8158), Paris, France

## Abstract

In any non-deterministic environment, unexpected events can indicate true changes in the world (and require behavioural adaptation) or reflect chance occurrence (and must be discounted). Adaptive behaviour requires distinguishing these possibilities. We investigated how humans achieve this by integrating high-level information from instruction and experience. In a series of EEG experiments, instructions modulated the perceived informativeness of feedback: Participants performed a novel probabilistic reinforcement learning task, receiving instructions about reliability of feedback or volatility of the environment. Importantly, our designs de-confound informativeness from surprise, which typically co-vary. Behavioural results indicate that participants used instructions to adapt their behaviour faster to changes in the environment when instructions indicated that negative feedback was more informative, even if it was simultaneously less surprising. This study is the first to show that neural markers of feedback anticipation (stimulus-preceding negativity) and of feedback processing (feedback-related negativity; FRN) reflect informativeness of unexpected feedback. Meanwhile, changes in P3 amplitude indicated imminent adjustments in behaviour. Collectively, our findings provide new evidence that high-level information interacts with experience-driven learning in a flexible manner, enabling human learners to make informed decisions about whether to persevere or explore new options, a pivotal ability in our complex environment.

## Highlights

• Study used instructions to modulate beliefs about informativeness of feedback.
• Reversal learning performance improved with perceived informativeness.
• Instruction-sensitive Hidden Markov Model provides good fit of behaviour.
• EEG recordings of feedback-related negativity (FRN) show modulation by instructions.
• Findings suggest reinforcement learning integrates experience with high-level beliefs.

## 1 Introduction

Humans and other animals use their ability to predict which action will lead to which outcome to choose appropriate actions and monitor their success. Occurrence of unexpected events can indicate incorrect or failed actions. However, in non-deterministic environments, unexpected events can happen for fundamentally different reasons: They may indicate true changes in the world and require adaptation, but sometimes they may instead reflect chance occurrence and should be discounted. To behave adaptively, an agent therefore needs to determine whether or not unexpected events indicate that a change in the environment has occurred. In other words, the agent must assess and integrate the event's *informative value*. Within this framework, the informative value of an unexpected event would be high, for example, if volatility in the environment was known to be high: unexpected events in volatile environments are more likely to reflect meaningful changes than unexpected events in stable environments. Thus, informative value is a parameter informed by a model of the world, which is at least partly dissociable from the unexpectedness of experienced events.

Learning from unexpected events, or prediction errors, is the focus of reinforcement-learning (RL) theories of adaptive behaviour. A core tenet of a major class of RL theories is that successful interaction with our environment depends critically on reducing the unexpectedness of events we encounter (Schultz et al., 1997, Sutton and Barto, 1990). Linking volatile environments to RL, previous work has shown that humans can use an experience-based estimate of volatility to adjust the rate at which they learn from unexpected feedback (Behrens et al., 2007). However, human learning does not rely solely on learning from direct experience: A fundamental human ability is to learn rapidly from explicit instruction, as instructions can provide a model of the world that helps to interpret events. Yet little is known about how instruction interacts with experience to shape behaviour (Cole et al., 2013).

The present experiments investigated the effect on trial-and-error learning of instructions that influence the perceived informative value of unexpected outcomes. We tested how a change in informativeness modulates adaptive behaviour and the neural correlates of feedback processing. Specifically, we investigated the impact of instructions about the environment (in terms of its volatility) or about feedback (in terms of its reliability) in a probabilistic reversal-learning task that required participants to integrate feedback to learn rules and adjust to rule changes.

In classical paradigms that focus on experience-based learning, informative value is so highly correlated with expectation and surprise that the two are often treated as isomorphic. Crucially, however, in the present experiments we dissociated effects of informative value from those of experience-based surprise: Instruction that response-outcome contingencies are volatile (i.e., likely to change) makes unexpected negative feedback more informative but at the same time less surprising, because learners should anticipate the occurrence of negative feedback indicating the need to adapt behaviour. Conversely, instruction that feedback is reliable (i.e., consistently indicative of choice accuracy) likewise makes feedback more informative, but makes unexpected negative feedback more surprising: If feedback is reliable, responses are more likely to yield expected (positive) feedback than unexpected (negative) feedback.

We tested the impact of instructions about environmental volatility and feedback reliability on adaptive behaviour and EEG correlates of feedback integration. We hypothesised that adaptation would be fast under volatility and reliability instructions, which should be evident in enhanced learning of correct responses following changes in the environment. In our EEG measures, we focused in particular on the feedback-related negativity (FRN) component as a marker of feedback processing, the stimulus preceding negativity (SPN) as a correlate of the anticipation of feedback, and the P3 as an index of feedback evaluation for immediate updating of action plans.

The FRN is observed as a rapid neural response (200–300 ms) following feedback presentation (Miltner et al., 1997, Gehring and Willoughby, 2002). A wealth of evidence has identified the FRN as a reward prediction error (RPE) signal of the kind proposed by RL theories (Holroyd and Coles, 2002): The FRN is typically observed following negative outcomes, with enhanced amplitude when negative outcomes are rare, or large in magnitude (Sambrook & Goslin, 2015; Walsh and Anderson, 2012). Our core hypothesis was that explicit instruction should change perceived informativeness of feedback, with consequent impact on feedback processing as reflected in the FRN. We expected the FRN to be increased when informativeness was high (under instructions suggesting volatility of the environment or highly reliable feedback), compared to conditions with lower informative value (under instructions suggesting stability of the environment or unreliable feedback). This hypothesis stands in contrast to existing characterisation of the FRN as reflecting the operation of a simple *model-free* RL system that learns purely from bottom-up experience (Holroyd and Coles, 2002, Walsh and Anderson, 2012), an interpretation supported by evidence that the component is strikingly insensitive to valid instruction about response-outcome associations (Walsh and Anderson, 2011). Such an RL account would predict that an increase in FRN amplitude following unexpected events would be unaffected by instructions that modulate informativeness.

The account of adaptive behaviour we adopt assumes that learning relies on explicit, structured internal models of the environment (Botvinick and Weinstein, 2014) and that the informative value of feedback, derived from this model, is integrated into learning and modulates neural correlates of feedback-processing. This framework suggests that processing of the environment is not a reactive process, but is instead actively guided by higher-order expectations. This conclusion would be consistent with recent findings and computational simulations indicating that estimates of uncertainty and volatility have partly independent effects on learning from feedback (Behrens et al., 2007, O’Reilly, 2013; Yu and Dayan, 2003; Mestres-Misse et al., 2016), and correspondingly have dissociable effects on the FRN (Bland and Schaefer, 2011). The latter finding is also consistent with an account of the FRN suggesting that it reflects an index of the demand of cognitive control; the demand for cognitive control is higher when information accumulates indicating the need for behavioural adaptation (Cavanagh and Frank, 2014).

We hypothesised that top-down modulation of the learning process would become further apparent in dynamic sampling of information according to its anticipated informative value. We therefore measured the SPN, a slow-wave potential observed prior to the presentation of feedback that provides useful information on task performance (Brunia, 1988, Morís et al., 2013). We expected a larger SPN amplitude under instructions suggesting high compared to low feedback informativeness.

The third EEG component of interest was the P3, which occurs after feedback presentation and is associated with the evaluation of feedback (Polich, 2007) and immediate behavioural responses (Chase et al., 2011). We expected to replicate Chase et al.'s (2011) finding that P3 amplitude is predictive of participants’ behaviour on the following trial, being enhanced prior to behavioural switches, and thus signifying the decision to adapt to the environment. In contrast to the FRN, which is associated with the integration of information in learning and was hence expected to scale with informative value, we expected the P3 to be more closely tied to the subsequent action and to reflect behaviour on the next trial independent of instructions.

## 2 Methods

### 2.1 Participants

Thirty-three participants took part in Experiment 1, 16 in Experiment 1a (7 female) and 17 in Experiment 1b (11 female). Average age in both parts of Experiment 1 was 21.5 years (18-30). Data from 5 participants were excluded from the final analysis, 4 because of excessive noise in the recordings, 1 because the participants failed to reach an accuracy level within 2-standard deviations of the population's mean performance.

Seventeen participants took part in Experiment 2 (7 female), with an average age of 22.0 years. 2 datasets had to be removed, one because of excessive noise, and one because the participant failed to reach an accuracy level within 2-standard deviations of the population's mean performance. All participants were right handed, had normal or corrected-to-normal vision, reported no history of neurological or psychiatric illness and gave written informed consent. They received monetary compensation for participation (£10/hour), but no performance-related bonus. The local ethics committee approved all procedures.

### 2.2 Stimuli and task

Both experiments used the same novel task, an instructed probabilistic reversal-learning paradigm. This task required participants to learn a new stimulus-response mapping in each block and to adapt this mapping if an unannounced rule reversal occurred. Participants were instructed to pay attention to the feedback to learn which of two possible stimulus-response mappings was correct. They were instructed that feedback was probabilistic and that a single rule reversal per block was possible. They were encouraged to keep paying attention to the trial-by-trial feedback throughout the block to detect any rule change that occurred. Prior to the main experiment, participants completed two practice blocks of the task outside the EEG booth and were allowed to ask questions. The experiments were run with the Psychophysics Toolbox version 3 (Brainard, 1997) in Matlab 2009b (The Mathworks, Inc., 2009) on a Windows PC attached to a 20 in. monitor at a resolution of 1024×768 and a refresh rate of 75 Hz. We measured response accuracy and reaction times during the main experiment for further behavioural analyses.

### 2.3 Experiment 1

**Fig. 1:**
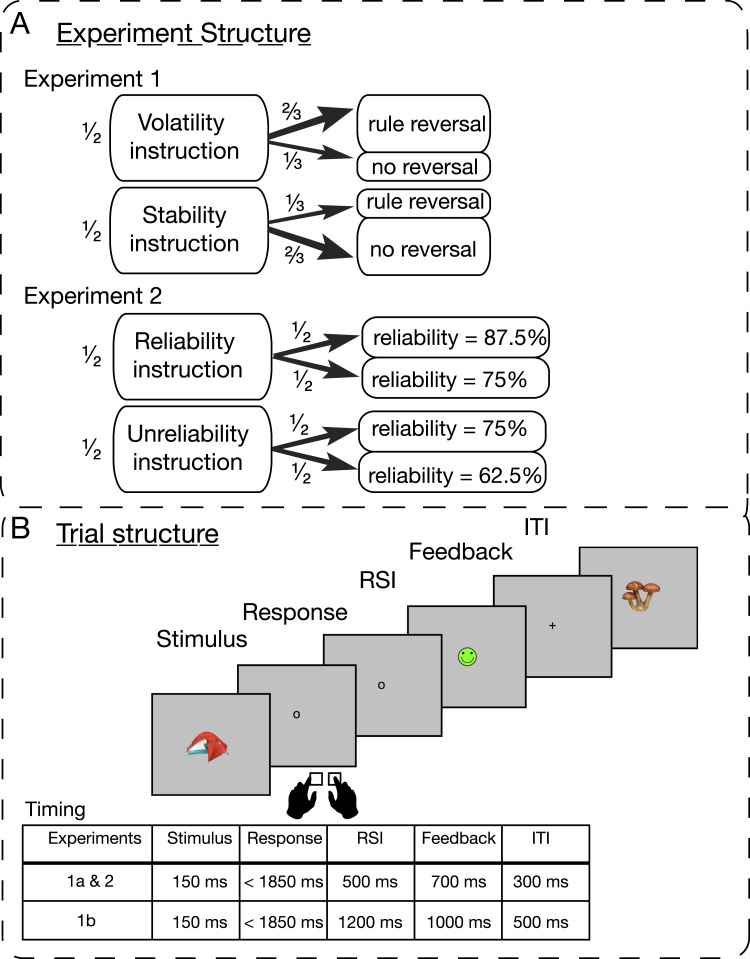
Paradigm setup. A: in Experiment 1, half of the blocks were instructed to be volatile, and the other half of the blocks were instructed to be stable. Following volatility instructions, the task rules reversed in 2/3 of the blocks. Following stability-instructions, rules only reversed in 1/3 of the blocks. Rule reversals occurred half way through the blocks, which varied in length to make the timing of rule reversals unpredictable. In Experiment 2, two different instructions, one indicating reliable feedback, the other one indicating unreliable feedback were paired with three degrees of reliability. The outer two conditions create a plausible context for the conditions of instruction-effect comparison. The latter conditions were critical, with a fixed, intermediate level of objective feedback reliability (75%) but with varying instruction about feedback reliability. B: in both experiments, participants had to respond to two different images per block, one of which required a left-hand response and the other one a right-hand response. Participants had to learn this mapping from the probabilistic, trial-wise feedback. (For interpretation of the references to colour in this figure, the reader is referred to the web version of this article.)

Each block started with a written instruction displayed on the screen. In Experiment 1, participants were instructed about the volatility of the environment (Fig. 1). Participants received the instruction: “The rules in this block will probably change” (volatility instruction) in half of the blocks, and the instruction “The rules in this block will probably remain stable” (stability instruction) in the other half. Rule reversals occurred in 2/3 of the volatility-instruction blocks and 1/3 of the stability-instruction blocks, with these probabilities made explicit to the subjects. The use of probabilistic instructions ensured that participants had to pay attention to the feedback and be engaged with the task regardless which instruction they had received. It also allowed us to measure the behavioural effects of instructions on adaptation. Because there was at most one rule reversal per block, we were able to measure the effects of instructions over a large number of trials, i.e., all trials that preceded the rule reversal. For all blocks in the experiment, pre-rule reversal trials differ in no parameter other than instruction. In each trial, participants had to press one of two keys (‘f’ and ‘h’ on a standard keyboard) with their left or right index finger in response to the image of a familiar object on the screen (Fig. 1, for a detailed description). The images were scaled so that they did not exceed 150 pixels in either width or height. There were two objects in each block, and new objects appeared in each block. A left-hand keypress was the initially correct response for one of the objects, and a right-hand keypress was the correct response for the other. Participants could only determine this initial mapping using feedback in a trial-and-error approach. Feedback contingencies were probabilistic, specifically being contingent on the correctness of the response in 75% of all trials: If participants implemented the correct mapping, they received positive feedback (a green smiley) in 75% of the trials and negative feedback (a red sad face) in 25% of the trials. For incorrect responses, participants received negative feedback in 75% of the trials and positive feedback in 25% of the trials. Failures to respond within a time limit of 2000 ms from stimulus onset were followed by a white, crossed-out face. Participants were told about the probabilistic feedback and knew that they had to integrate feedback over a number of trials to learn the correct mapping and to detect rule reversals.

**Fig. 2:**
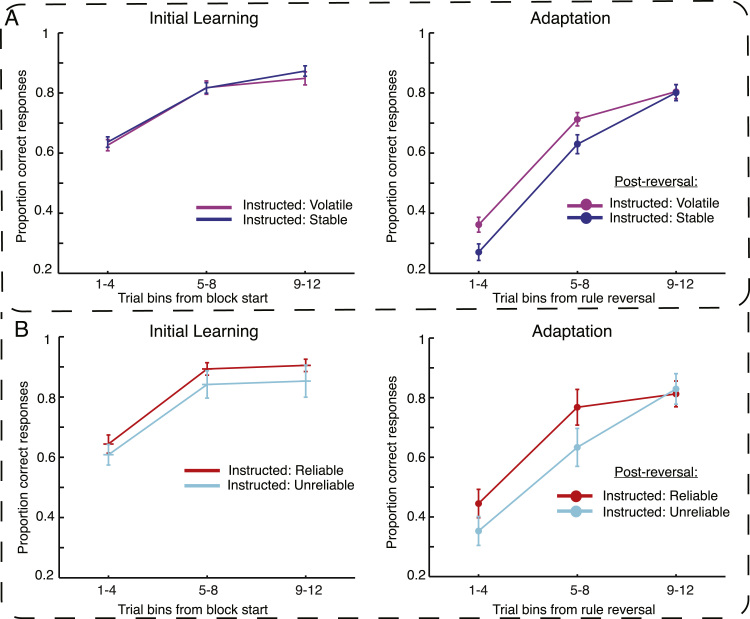
Learning rates. Pattern of behavioural accuracy in experiment 1 (A) and experiment 2 (B). Percent correct responses are shown for bins of 4 trials from the start of each block (left panels), or the switch trial (right panels), respectively. A: Participants learned as fast under volatility instruction (pink) as under stability instruction (blue), as evident from virtually identical accuracy in the three bins covering the first 12 trials. However, there was a clear effect of volatility instruction on adaptation behaviour, as evident in lower accuracy for the first few trials following the switch under stability compared to volatility instructions. B: Participants learned faster and performed slightly better under reliability (red) compared to unreliability instructions (cyan). Likewise, adaptation was faster following reliability compared to unreliability instructions. All error bars display standard-error of the mean. (For interpretation of the references to colour in this figure legend, the reader is referred to the web version of this article.)

Block lengths varied randomly between 25, 33, and 41 trials, and rule reversals occurred half-way through the respective blocks, i.e., on trial 13, 17, or 21. Block-length was counterbalanced across conditions. The symmetric setup within blocks has two advantages: First, it minimised participants’ ability to build an expectation about when rule reversal would occur, which otherwise could have helped them to decide whether an unexpected negative feedback was more likely to be caused by a rule reversal (Fig. 2). Second, having as many trials before and after the rule reversal increased participants’ motivation to adapt to rule changes, and also allowed us to run statistical analysis on conditions with an equal number of trials. Performance in the pre-rule reversal phase of volatility-instructed blocks was compared with the same number of trials from the first half of stability-instructed blocks. Thus, trial numbers and trial-position in the block were kept constant across comparisons. The same approach was taken to post-rule reversal analyses of accuracy: This analysis compared performance in trials from the second halves of the rule reversal blocks to trials from the second halves of non-reversal blocks, again achieving equal trial-numbers and comparable trial-histories thanks to the balanced setup of block lengths across conditions. Participants received feedback on percent correct responses after each block during a short, self-paced pause. Experiments 1a and 1b differed critically in the interval separating the response on a given trial and subsequent feedback. In Experiment 1a this interval was 500 ms. In Experiment 1b, we lengthened this interval to 1200 ms to enable us to measure slow preparatory potentials preceding feedback delivery. Experiment 1a had 36 blocks and experiment 1b, owing to the longer response-feedback interval in each trial, had 27 blocks (Fig. 1).

#### 2.3.1 Behavioural analysis

Behavioural analysis focused on two aspects of behaviour: We first wanted to establish that, prior to a potential rule reversal, participants learned equally well under the two instruction conditions (initial acquisition). To assess this we calculated participants’ average accuracy in the first half of each block, and also the average number of trials from the start of each block before participants first repeated the correct rule on two successive trials (a key indication that they had established this rule, and were now in a mode of deliberate exploitation as opposed to explorative, or guessing behaviour). Correct responding was defined as applying the currently correct rule, not as receiving positive feedback (which occurred probabilistically). The second focus of the behavioural analysis targeted the impact of instructions on adaptation after rule reversals. Here, we used the same two performance measures as in the first analysis, but focused on the second half of the blocks in which a rule reversal occurred to assess the influence of instructions. For this post-reversal phase, we expected participants to show reduced accuracy in stability-instructed blocks. We additionally calculated the probability with which participants would reverse their response mapping following surprising feedback as a further indication of adaptive modulation of behaviour by instructions.

#### 2.3.2 Task design – expectation of negative feedback

A key feature of our design is that it controls for the relative frequency of negative and positive feedback and thereby the effects of low-level unexpectedness. At the same time, it independently manipulates the surprise associated with negative feedback and its informativeness in a given instruction condition. If performance prior to rule reversals is comparable between the conditions (volatility-instructed and stability-instructed blocks)—as will later be shown to be the case—the two conditions will have the same frequency of negative feedback in the trials that enter the EEG analysis. Therefore, simple frequency effects could not explain any differences observed in the EEG correlates of feedback processing. Meanwhile, different levels of accuracy between conditions over the entire block length, i.e., including the second halves of the blocks (which are not entered into the EEG analysis) would be expected to modulate participants’ expectations of negative or positive feedback associated with an instruction. Specifically, this higher-level expectation should make negative feedback less surprising in volatility-instructed blocks compared to stability-instructed blocks. To foreshadow this important feature of our experiment, we found that the probability of receiving negative feedback was indeed significantly higher in volatility-instructed than in stability-instructed blocks (*t*(27)=5.22, *p*<0.01, two-tailed), owing to an increase of incorrect responses *following* rule reversals. Unexpectedness of negative feedback was therefore lower under volatility instructions than stability instructions for a learner who took instructions into account. In sum, negative feedback under volatility instructions was on average more informative but was also on average less surprising than negative feedback under stability instructions, thus de-confounding informativeness and surprise measures, which typically co-vary.

### 2.4 Experiment 2

In this experiment, we tested whether effects of perceived informativeness on feedback processing would generalise to instructions that do not inform on volatility of the mapping but that directly concern the feedback itself. Here, the pre-block instruction concerned the *reliability of feedback*. Higher (instructed) reliability made feedback more informative than lower (instructed) reliability. In half of the blocks, participants were instructed: “The feedback in this block will be reliable” (reliability instruction). In the other half, participants were instructed: “The feedback in this block will be unreliable” (unreliability instruction).

These two types of instructions preceded blocks with *three* different degrees of reliability. One quarter of all blocks had highly reliable feedback (87.5% contingent on correctness of the response). These blocks were always preceded by the reliability instruction. A second quarter of all blocks had considerably less reliable feedback (62.5% contingent on correctness of the response). These blocks were always preceded by the unreliability instruction. The remaining blocks were of intermediate feedback reliability, which was the same as implemented in Experiment 1 (75% contingent on correctness of the response). Half of these blocks with intermediate reliability (1/4 of all blocks) were preceded by the reliability instruction, whilst the other half was preceded by the unreliability instruction (Fig. 1). These latter two block types (fixed intermediate level of reliability, two types of instructions) are the crucial blocks for analysis, which allowed us to test for instruction effects comparable to Experiment 1.

The task was the same probabilistic reversal-learning task as in Experiment 1. A single reversal occurred in 3/4 of the blocks (each reliability condition appeared 8 times over the entire experiment, creating an equal number of reversals per reliability condition). Block lengths were set to 33 trials and the single rule reversal occurred equally often on trial 9, 17, or 25. This design choice differed slightly from the setup in Experiment 1 but preserved the core characteristics: First, setting the average rule reversal trial to the middle of the block (trial 17), and at least 9 trials before the end of the block again ensured that participants had the motivation and opportunity to adapt to the new rule. Second, as the reliability levels can be realized as proportions of 8 trials (highly reliable: 7/8 trials contingent, intermediate reliable: 6/8 contingent, highly unreliable: 5/8 contingent), locating the switch after multiples of 8 trials allowed us to keep the reliability in the run-up to the rule reversal and post rule reversal evenly distributed. Lastly, not exceeding 33 trials in length (which is the average trial-length in Experiment 1)—even after late rule reversals—increased design efficiency, as the EEG analyses again focused on the pre-rule reversal phase of each block. Participants were again explicitly informed about the rule reversal probability. Importantly, however, they did not know that more than two degrees of reliability existed. They received feedback on the percentage of correct responses in each block during a short, self-paced pause after each of the 32 blocks.

In summary, the difference in informativeness by instruction in this experiment again relates to the probability that an unexpected negative event was indicative of a change in the rules. Over all blocks of the experiment (including the truly more reliable and truly more unreliable feedback blocks), this probability was higher following reliability instructions than unreliability instructions.

#### 2.4.1 Behavioural analysis

Analysis focused on the conditions that varied in instructed reliability but in fact had the same feedback contingency. Our analyses implemented the same tests as the analysis of Experiment 1. The relevant markers of behaviour were percent correct responses in the part of the block preceding a rule reversal and trials-to-repetition of the initially correct mapping as measures of initial acquisition and performance (which were both expected to be unaffected by instructions, as in Experiment 1). Further, we again measured percent correct performance and trials-to-repetition after rule reversals to assess the effects of instructions on adaptation (which were expected to differ by instruction). We used probability of reversing the mapping following surprising feedback as an additional measure of instruction effects on adaptive behaviour.

#### 2.4.2 Task design – expectation of negative feedback

As will be shown later, participants’ performance (and therefore number of negative feedback events) prior to rule reversals did not differ reliably between blocks of equal feedback reliability but different instructions. However, overall, participants received more negative feedback in blocks that were instructed to be unreliable, as these include blocks in which *feedback was indeed unreliable,* which has negative effects on performance. To summarise, in contrast to Experiment 1, participants should be more surprised by negative feedback in the same condition under which feedback was considered to be more informative, i.e., in the blocks that were instructed to be reliable.

### 2.5 EEG recordings

Participants sat in an electrically shielded, sound attenuating booth to minimise artefacts in the EEG recordings. A Neuroscan Synamps2 system (10 GΩ input impedance; 29.8 nV resolution; Neuroscan, El Paso, TX, USA) was used to record EEG data from 32 Ag/AgCl electrodes mounted in an elastic cap at locations FP1, FPZ, FP2, F7, F3, FZ, F4, F8, FT7, FC3, FCZ, FC4, FT8, T7, C3, CZ, C4, T8, TP7, CP3, CPZ, CP4, TP8, P7, P3, PZ, P4, P8, POZ, O1, OZ, and O2. Six additional external electrodes were attached to the outer canthi of the left and right eyes, above and below the right eye to measure electro-oculograms (EOGs), and to the left and right mastoids. Electrode recordings were referenced to the right mastoid. All electrode impedances were kept below 50 kΩ. EEG data were recorded at a sampling rate of 1000 Hz. Online high-pass filtering was implemented for experiment 1a and 2 at 0.1 Hz. Online high-pass filtering was avoided for experiment 1b to allow us to measure slow-wave EEG activity preceding feedback delivery.

### 2.6 EEG data analysis

In both experiments, the core question addressed was whether instructions that changed participants' belief about the informativeness of specific feedback would modulate feedback processing. Our analysis focused primarily on the amplitude of the FRN, a negative-going EEG waveform following feedback onset that is typically associated with the prediction-error learning signal (Sambrook and Goslin, 2014, Hauser et al., 2014, Holroyd and Coles, 2002). We hypothesised that informativeness would impact not only processing of presented feedback, but also anticipation of feedback, a signature of a learning process that involves dynamic sampling of information. We therefore assessed whether the amplitude of the stimulus-preceding negativity (SPN) prior to feedback onset in Experiment 1b would be increased under reliability instructions. Because the SPN is associated with the anticipation of informative feedback (Kotani et al., 2003), we considered an increase in amplitude as a marker of preparation for information sampling. As a marker of later cognitive evaluation of feedback and strategic modulation (Chase et al., 2011; see Polich, 2007, for review), we measured the P3 component that occurs a few hundred milliseconds after feedback delivery. Finally, to assess whether any observed modulations of the FRN, SPN and P3 might be driven by low-level changes in visual attention to feedback, we analysed N1 and P1 potentials evoked by feedback onset. Both components are strongly associated with directed attention towards an external stimulus, be it in the auditory (Näätänen and Picton, 1987) or visual domain (Luck et al., 2000, Eimer, 2014). Increased P1 and N1 amplitudes are taken to reflect increased attention towards the stimulus, such as may be expected, for example, as a correlate of increased task engagement.

Eye-blink correction was conducted using an independent components analysis approach via the EEGLab toolbox for Matlab (Delorme and Makeig, 2004) in Experiment 1a, and using a regression approach (Semlitsch et al., 1986), implemented in Scan 4.5 (Neuroscan, El Paso, TX, USA) in Experiments 1b and 2. After epoching the data (details below), trials with voltage differences >100 µV were discarded. All analyses were performed on data down-sampled to 250 Hz. Offline filtering was achieved with a Hamming-window synchronised finite impulse response function, as implemented in EEGLab (Widmann and Schröger, 2012). For the FRN analysis, P3 analysis, and analysis of N1 potentials in Experiments 1 and 2, data epochs were extracted from −500 ms prior to feedback onset to 1500 ms post feedback onset. EEG data were offline high-pass filtered at 0.1 Hz and low-pass filtered at 24 Hz. We baseline corrected each epoch to a time window from −200 ms pre feedback onset to −100 ms pre feedback onset in both experiments.

#### 2.6.1 Experiment 1

##### 2.6.1.1 FRN analysis

The FRN was estimated using an average-base to peak measure (Yeung and Sanfey, 2004, Chase et al., 2011). We averaged voltage measures over a fronto-central cluster comprising the electrodes: F3, FZ, F4, FC3, FCZ, FC4, C3, CZ, C4 (voltage topographies in Fig. 4) and calculated the lowest voltage in a time window from 240 ms to 280 ms post feedback onset, and the highest voltage in the preceding and following positive-going components (time windows: 160 ms to 220 ms post feedback onset and 300 ms to 420 ms post feedback onset, respectively). The most negative value was then subtracted from the mean of the two positive peaks to give FRN amplitude. If the highest point was on the edge of a peak window, the window was gradually widened until the highest point no longer fell on the edge (Chase et al., 2011). Results with parallel analyses using quantification of the FRN as simple base-to-peak amplitude did not differ materially from those reported below.

FRN analysis in both experiments included only trials in which participants applied the currently correct rule, preceding the rule reversal. In Experiment 1, this included the trials from the first half of all blocks during which a rule reversal occurred and the trials from the first half of all the length-matched blocks that contained no rule reversal. Importantly, these trials differed only with regard to the instruction, but were otherwise identical. We thus ensured that equal numbers of pre-switch trials in volatility and stability-instructed blocks entered the analysis. Error trials were excluded from the analysis, as participants’ feedback expectations are unclear in these trials. The FRN analysis therefore contained 4 categories of feedback: positive vs. negative feedback after correct responses under stability instruction, and positive vs. negative feedback after correct responses under volatility instruction. Average single-subject FRN amplitudes were entered into a repeated-measures ANOVA with the factors INSTRUCTION (stability/volatility) and VALENCE (positive/negative). In a second step, we included EXPERIMENT VERSION (a or b) as a between-subject factor in a 2×2×2 repeated-measures ANOVA to rule out that duration of the response-feedback interval had any influence on the established FRN effect.

##### 2.6.1.2 SPN analysis

To test whether the amount of expected informative value of the feedback (Brunia, 1988, Kotani et al., 2003, Morís et al., 2013) would lead to an active preparation for more relevant events, we measured the stimulus preceding negativity (SPN) between participants’ responses and feedback onset. The response-feedback interval in Experiment 1b was increased to 1200 ms to make measuring this slow-wave potential possible.

The EEG data were epoched to response onset, with epochs beginning −500 ms prior to response onset and ending 500 ms post feedback onset. The EEG data were high-pass filtered at 0.05 Hz and low-pass filtered at 24 Hz. The soft high-pass filter leaves the type of slow-wave potential that we were interested in intact while preventing artefacts from slower voltage drifts. We baseline corrected epoched data to a time window from 200 ms after response onset to 300 ms after response onset. This analysis followed the measures taken in a recent publication which shows that the SPN tracks the value of feedback over the course of learning (Morís et al., 2013): SPN amplitude was measured as the mean amplitude in three different pre-feedback time windows 1: −600 ms to −400 ms, 2: −400 ms to −200 ms, and 3: −200 ms to feedback onset. Data were extracted from an electrode cluster spanning: FC3, FCZ, FC4, C3, CZ, C4, CP3, CPZ, and CP4. Because the SPN is typically larger over the right than the left hemisphere, and amplitude increases gradually, we implemented a 2×3×3 repeated-measures ANOVA, with the factors INSTRUCTION (volatility/stability), TIME (window: 1/2/3) and LATERALITY (left/central/right).

##### 2.6.1.3 P3 analysis

Two main questions motivated the P3 analyses: First, we wanted to establish whether the P3 would show a comparable instruction effect to the FRN. We therefore mirrored the FRN analysis for the P3. Single-subject P3 amplitudes were measured as the maximum voltage in condition-averaged EEG waveforms within a time window 300 ms to 420 ms post feedback onset (same as the second peak in the FRN measure), across a centro-parietal electrode cluster containing the electrodes: CP3, CPZ, CP4, P3, PZ, P4, and POZ (cf. posterior cluster in Chase et al. (2011), voltage topography maps in Fig. 5). Average single-subject P3 amplitudes were entered into the repeated-measures ANOVA with the factors INSTRUCTION (stability/volatility) and VALENCE (positive/negative).

Second, we aimed to replicate evidence for a close link between the P3 and behavioural decisions as described by Chase et al. (2011), who showed that P3 amplitude predicts reversal behaviour on a trial-by-trial basis. We therefore measured P3 amplitude as described above in trials with negative feedback outcomes within the first half of all blocks and tested in a repeated-measures ANOVA with the factors NEXT TRIAL BEHAVIOUR (repeat/reverse) and INSTRUCTION (stability/volatility) whether P3 amplitude would be significantly larger preceding trials in which participants reversed their behaviour, compared to repetition trials.

##### 2.6.1.4 Visual potentials: P1 & N1

We analysed the P1 and N1 potentials to assess whether any between-condition differences in EEG activity might reflect differences in low-level attention to the feedback, which could hint, for example, at decreased task-engagement in a given condition. We estimated the P1 amplitude as the maximum amplitude across a parietal cluster of electrodes in the standard time window of 60 ms to 100 ms post feedback onset. The cluster of electrodes was chosen in a data-driven fashion by assessing the electrodes that reached the highest mean amplitude in the 4 conditions. This yielded a parietal cluster comprising P7, P3, PZ, P4, P8, POZ, O1, OZ, and O2. We also estimated the parietal N1 potential as the minimum voltage across the same electrodes as the P1 in a time window from 140 to 200 ms after feedback onset. Amplitudes of the P1 and N1 potentials were then entered into separate repeated-measures ANOVAs with the factors INSTRUCTION (volatility/stability) and VALENCE (positive/negative) to mirror the FRN analysis.

#### 2.6.2 Experiment 2

All components of interest were quantified in the same manner as for Experiment 1. A crucial design difference between the two experiments was that Experiment 2 included four block types rather than two: It included two block types with equivalent feedback reliability (75%) but differing instructions, and two blocks differing in objective feedback reliability (87.5% vs. 62.5%). Our core analyses contrasted the first two block types, where feedback contingencies were objectively identical but subjective expectations differed. These analyses of the FRN, P3, and N1 and P1 used repeated-measures ANOVAs with the factors INSTRUCTION (reliable/unreliable) and VALENCE (positive/negative), and included all correct trials preceding a rule reversal. For comparison with the pure-instruction effects we observed, and with prior studies of the FRN that have manipulated objective feedback reliability, we also report FRN analyses that contrast blocks differing in objective feedback reliability (87.5% vs. 62.5% reliability). For this analysis we entered FRN amplitude measures into a repeated-measures ANOVA with the factors CONDITION (reliable/unreliable) and VALENCE (positive/negative).

## 3 Results

### 3.1 Experiment 1

#### 3.1.1 Experiment 1 – behavioural analysis

Experiment 1 investigated the effect of instructions about the volatility of the environment on feedback processing. To compare the neural correlates of feedback processing, it was important first to show that volatility instructions did not disrupt initial learning of the mapping. All statistical analyses, if not stated otherwise, are two-tailed, paired-sample *t*-tests, with an alpha-level of 0.05.

##### 3.1.1.1 Experiment 1 – Initial learning

To test for potential effects of instructions on learning of stimulus-response mappings, we compared accuracy during the first halves of all blocks (which differ only in terms of instructions). As expected, there were no reliable differences between the instruction types on performance accuracy (*t*<1): Mean accuracy was 80% for stability instruction blocks (Standard-error of the mean (*SEM*)=1%) as compared with 79% (*SEM*=1%) in volatility-instructed blocks. As a related measure, we assessed whether instructions changed how efficiently participants integrated feedback to acquire the initial mapping. We therefore measured how many trials it took participants to repeat the correct mapping, measured from the first trial of each block. Again, we found no significant differences between instruction conditions, with 2.77 (*SEM*=0.13) vs. 2.72 (*SEM*=0.09) trials, respectively (*t*<1). Participants received negative feedback on average on 37% (*SEM*=1%) of trials during the first half of volatility instructed blocks and on 34% (*SEM*=6%) of trials in the first half of stability instructed blocks. The difference was not significant (*t*<1). These findings are relevant in interpreting analyses of the FRN, which is usually described as a correlate of frequency-based unexpectedness. Informativeness can only be separated from low-level frequency effects if participants experience the same amount of surprising negative feedback under both instruction conditions during the part of the blocks that enter the FRN analysis. The initially equivalent performance shows that this was the case.

##### 3.1.1.2 Experiment 1 – the effect of instructions on adaptation

Clear effects of instructions became apparent when we compared behaviour in the second halves of the blocks. Following a rule reversal, participants reached higher accuracy levels under volatility than stability instructions (68%, *SEM*=1%, vs. 64%, *SEM*=1%; *t*(27)=2.5, *p*<0.01). This performance difference was brought about by faster adaptation to expected than non-expected rule reversals, revealed by significantly fewer trials-to-repetition after rule reversal under volatility instruction than stability instructions (*4.7*, *SEM*=0.25, vs. 5.69, *SEM*=0.27, respectively; *t*(27)=3.61, *p*<0.01). More evidence for the role of instructions, even in the absence of real changes in the environment, came from a comparison of performance in terms of percentage correct responses for the second halves of the blocks where no reversal occurred. Participants performed worse when they expected rule reversals than when they did not (*t*(27)=3.68, *p*<0.01).

These differences in adaptation rate across instruction conditions were apparent in the earliest blocks of the experiment, and did not reliably increase in amplitude across blocks. The average difference in trials-to-repetition between the first rule reversal under volatility instructions and the first reversal under stability instructions was 2.32 trials; this difference is statistically significant in a paired-samples *t*-test *t*(27)=3.07, *p*=0.0024. The effect size is re-assuring given that this analysis relies on single block of data per subject and condition: Cohen's *d*=0.78. The difference between instructions for the last block with a rule reversal in each respective instruction condition was 1.39, a difference that was also statistically significant in a paired-samples *t*-test *t*(27)=1.82, *p*=0.039; Cohen's *d*=0.49. There is no statistically significant effect of block when we compare the difference in trials-to-repetition by instruction conditions in the first and last block of each respective condition (*t*(27)=0.96, *p*=0.34; Cohen's *d*=0.25). Taken together, these results suggests that observed differences across conditions reflect participants’ ability to adjust their learning flexibly and rapidly according to the instruction provided, rather than reflecting long-term learning (i.e., based on the experience of prior blocks with differing instructions).

To test whether the comparative advantage in adapting to a new rule under volatility instructions was caused by more exploratory behaviour following surprising feedback under volatility than stability instructions (in the absence of actual rule reversals), we compared across instruction conditions the proportion of trials in which participants reversed the present mapping following a surprising negative outcome. As expected, we found a significant effect of instruction on the probability of switching to the alternate mapping following negative feedback in the first half of blocks (*t*(27)=2.08, *p*<0.05), with a larger propensity to switch in volatility instruction blocks than stability instruction blocks (21% vs. 19%). The same comparison did not yield significant differences in the second half of blocks following actual rule reversals (*t*<1), presumably because participants understood that rules would only reverse once per block.

In sum, these analyses showed that participants used instructions to improve their behaviour and, crucially, that the rate of negative feedback between different instructions does not increase low-level unexpectedness of negative feedback under volatility instructions.

##### 3.1.1.3 Experiment 1 – no differences in model-free negative RPEs

The preceding analyses demonstrate that, at an aggregate level, negative feedback was less surprising following volatility instructions than stability instructions (numerically so in the first halves of blocks, and reliably so considering both block halves). As an additional measure to further rule out the possibility that differences in FRN amplitude between instruction conditions in our paradigms may be conflated with differences in the low-level unexpectedness of negative feedback at a trial-by-trial level, we quantified instruction-blind unexpectedness by implementing a standard model-free RL learning algorithm. We applied this algorithm to calculate trial-by-trial reward prediction errors (RPEs) in all blocks (learning rate=0.5) according to the actual sequence of stimuli, responses and outcomes experienced by each participant. As with our EEG analyses, we focused on RPEs in first half of each block, where blocks differed solely in terms of instructions. Comparing the average RPE size (for signed, negative RPEs, which correspond to unexpected negative events) across instruction types, we found no significant difference (*t*<1). As intended, this shows that an instruction-blind reinforcement-learning algorithm that treats unexpected feedback identically under different instruction conditions cannot explain the predicted differences in FRN amplitude.

##### 3.1.1.4 Experiment 1 – Hidden Markov Model shows advantage of instruction sensitivity

To test formally whether an artificial learner that is sensitive to instructions would capture behaviour in the task, we compared two Bayesian Hidden State Markov Models (HMM; Ghahramani, 2001; Hampton et al., 2006). This family of models has been shown to outperform reinforcement learning models in explaining reversal learning in previous work (Hampton et al., 2006) and we followed this approach closely in the construction of our basis model. The models that we tested against each other differed with regard to whether they were instruction blind (basis model), or instruction sensitive (instruction model). Thus, rather than compare RL and HMM algorithms as presented by Hampton et al., (2006), we aimed to establish an advantage of an instruction-sensitive compared to an instruction-blind learner, within a class of models already known to be successful in reversal-learning. Decisions to reverse or persist with a mapping were based on a trial-by-trial estimate of uncertainty in the environment (formalised as entropy, Shannon, 1948; please refer to the Supplemental material for a full description of the models).

**Fig. 3:**
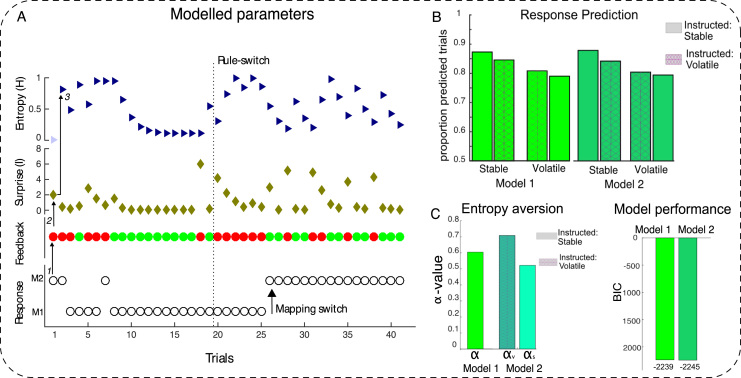
HHM. A: modelled parameters. Participants gave a response on every trial (1), either implementing mapping 1 or mapping 2, according to which one they believed reflected the correct mapping at that time. In this example, the required mapping (i.e. the state of the world) switches after 19 trials; the participant needs 6 trials to adjust to this switch. Each response was paired with feedback in the form of positive (green) and negative (red) smileys (2). The information of the feedback becomes integrated with the prior of the implemented mapping being correct (initially at 0.5), and the information (surprise) associated with this outcome is captured in I. Unexpected negative feedback leads to an increase in the Surprise parameter I; during a series of negative feedback outcomes towards the implemented mapping, this value decreases as the prior probability of the correctness of the implemented mapping decreases, too. Entropy (H) reflects the uncertainty that results from an accumulation of informative outcomes, and thus the uncertainty at the beginning of the respective next trial (3). B: the HMM switches the mapping when an individually fitted entropy-aversion parameter (alpha) is crossed. An instruction-blind model (model 1), assuming the same entropy-aversion score for all types of blocks (displayed in c), leads to slightly lower percent correctly predicted trials at the level of the individual, than an instruction-sensitive model (model 2). C: the individually fitted alpha values explain why participants switch faster in blocks with volatility instruction (patterned bars) – participants displayed significantly greater entropy aversion under volatility compared to stability instructions; The BIC model comparison yields a difference of approx. 6 suggesting a positive advantage of the instruction-sensitive over the instruction-blind model (Kass & Raftery, 1995). (For interpretation of the references to colour in this figure legend, the reader is referred to the web version of this article.)

As expected, model comparison using Bayesian information criterion (BIC) showed a positive (significant) advantage (Kass and Raftery, 1995) of the instruction-sensitive model (model 2) over the instruction-blind model. Further, the results of the instruction-sensitive parameter fitting (see supplement) suggested that participants were more averse to uncertainty under volatility than under stability instructions. In formal terms, the entropy avoidance parameter, α, was significantly larger across the group under volatility than under stability instructions (Mean *α*_v_=0.7 *SEM*=0.22; Mean *α*_s_=0.52, *SEM*=0.72 *t*(27)=3.22, *p*=0.003). Both models performed satisfactorily at >79% correctly predicted trials in all conditions (Fig. 3b). The presented models give a reasonable, albeit imperfect fit to the behavioural data. Which exact model will fit human behaviour best is a matter of ongoing research, but the comparison of these reasonably successful models suggests that artificial learners which compare experience with expectations about the environment, are better at explaining human behaviour than agents blind to this higher-order information.

#### 3.1.2 Experiment 1 – EEG analysis

##### 3.1.2.1 FRN modulation by volatility instructions

**Fig. 4:**
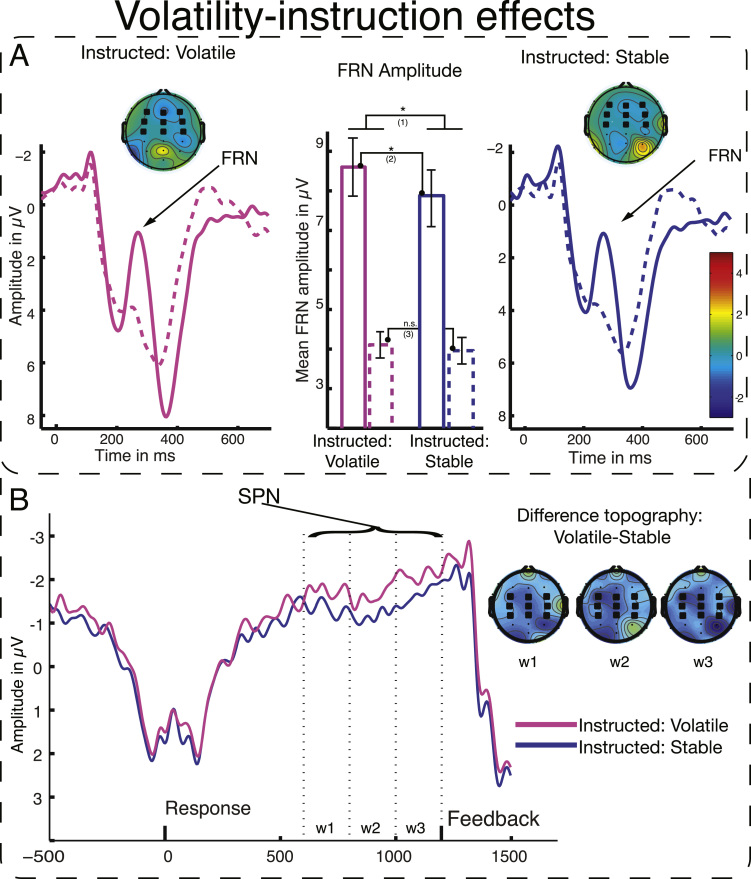
Modulation of ERPs by Volatility Instruction. A: time–voltage plots showing the FRN component following positive (dashed lines) and negative (solid lines) unexpected feedback under volatility (left panel) and stability (right panel) instructions. The bar graphs (middle panel) plot the average over individual amplitudes, showing the significant effect of instruction on amplitude (1), and the significant difference between FRN amplitude following unexpected negative events in the comparison of volatility-instructed and stability-instructed blocks (2). Voltage topographies show the difference between positive and (unexpected) negative feedback under the respective instruction conditions in the time interval between 200 ms and 310 ms post stimulus onset. B: The time–voltage plots for the SPN show that this negative pre-feedback component reached a higher amplitude (lower voltage) preceding feedback under volatility compared to stability instructions. W1-3 refers to the time-windows for analysis. Voltage topographies show the difference in raw voltage between volatility and stability instruction conditions in the last time window. Dark electrodes delineate clusters that entered the respective statistical analysis and correspond to the electrodes averaged in time–voltage plots. All error bars display standard-error of the mean.

The primary EEG analysis of Experiment 1 tested whether instructed volatility—which should increase informativeness of feedback events—would modulate FRN amplitude. We hypothesised that the neural response towards unexpectedness is modulated by the perceived informativeness of the event, and therefore that we would observe larger FRN amplitude under volatility compared to stability instructions. In line with this hypothesis, we found a main effect of INSTRUCTION (*F*(1,27)=5.36, *p*=0.030) in the predicted direction, with a larger FRN for feedback under volatility compared to stability instructions in the 2×2 repeated-measures ANOVA (Fig. 4). Further, we established a main effect of VALENCE (*F*_(1,27)_=34.74, *p*<0.001) with the typical pattern of a larger negative extent of the waveform for negative than positive feedback. There was no statistically significant interaction between the effects (*F*_(1,27)_=2.28, *p*=0.142). Investigating the main effect of instruction further in planned comparisons, we found that there was a significant difference in FRN amplitude following negative feedback under volatility instructions as compared to stability instructions: *t*(27)=2.55, *p*=0.016. However, the paired *t*-test for effects of instruction in positive feedback events failed to show a significant difference: *t*<1.

To assess whether differences in response-feedback interval affected the FRN, we ran an additional 2×2×2 repeated-measures ANOVA, including the between-group factor EXPERIMENT VERSION (1a/1b). We found no effect of this between-group variable (*F*<1) and no interaction of the between group variable with either of the two main effects (interaction with INSTRUCTION: *F*<1; interaction with VALENCE: *F*_(1,27)_=1.71, *p*=0.2). Finally, there was also no reliable three-way interaction between EXPERIMENT VERSION, INSTRUCTION, and VALENCE (*F*_(1,27)_=1.07, *p*=0.3).

##### 3.1.2.2 SPN modulation by volatility instructions

We expected instructions to change not only feedback processing, but also anticipation of feedback as it is reflected in the SPN. In a repeated-measures ANOVA with the factors INSTRUCTION, TIME, and LATERALITY, we established the predicted effect of INSTRUCTION (*F*_(1,13)_=7.01, *p*=0.02). The SPN reached greater (i.e., more negative) amplitude under volatility instructions than under stability instructions, a sign of increased preparation for feedback processing in this condition. We further established a significant effect of LATERALITY (*F*_(2,26)_=5.88, *p*=0.008), reflecting the typical right-hemisphere dominance of the SPN. The effect of TIME reached only marginal significance (*F*_(2,26)_=2.69, *p*=0.087), but there was a significant interaction between the TIME and LATERALITY (*F*_(4,52)_=3.1, *p*=0.023), because the difference between the right and left hemisphere in the amplitude of the negative deflection of the waveform increased over time.

##### 3.1.2.3 P3 modulation reflecting behavioural adaptation

**Fig. 5:**
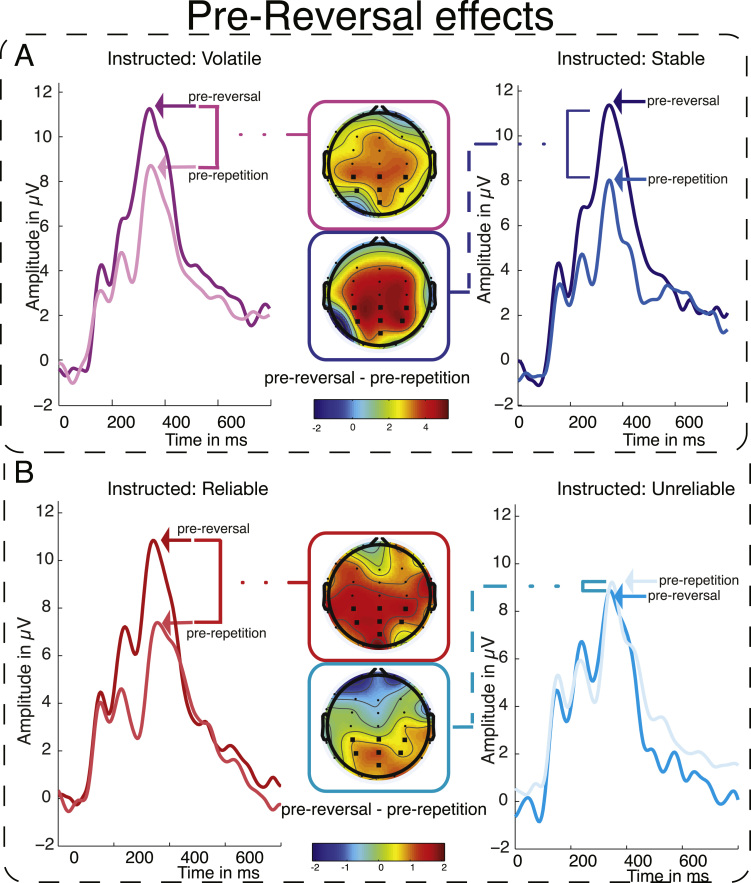
Reversal effects on P3 amplitude. A: Effects of behaviour on the next trial on P3 amplitude under volatility (left panel) and stability (right panel) instructions. The P3 amplitude was enhanced preceding reversals of the current mapping (dark lines), compared to repetitions of the ongoing mapping under both instruction conditions. B: Effects of behaviour on the next trial on P3 amplitude under reliability (left panel) and unreliability (right panel) instructions. There is a positive difference between trials preceding reversals compared to repetitions under the reliability instructions. A and B: Voltage topographies show the difference between trials preceding reversals and repetitions under the respective instruction conditions, dark electrodes delineate the cluster that entered the statistical analysis and underlies the time–voltage plots to either side.

A first analysis of the P3 assessed whether this component would show similar modulation by informativeness as the FRN and SPN. The results indicated not: For the P3 we found no reliable effect of INSTRUCTION (*F*_(1,26)_=2.8, *p*=0.102), but a significant effect of VALENCE (*F*_(1,26)_=7.8, *p*<0.01) with greater P3 amplitude following negative than positive feedback, and no interaction of INSTRUCTION and VALENCE (*F*<1). Our second analysis of the P3 focused on its relationship with behaviour on trials following negative feedback (cf. Chase et al., 2011). In a 2×2 repeated measures ANOVA with the factors NEXT TRIAL BEHAVIOUR (reversal or repetition) and INSTRUCTION, we found a significant effect of NEXT TRIAL BEHAVIOUR (*F*_(1,26)_=33.79, *p*<0.001), with greater P3 amplitude following negative feedback that led to reversals of behaviour (Fig. 5). However, in this analysis we found no main effect of INSTRUCTION (*F*<1) and no interaction between NEXT TRIAL BEHAVIOUR and INSTRUCTION (*F*_(1,26)_=1.95, *p*=0.17). We thus established that P3 amplitude was relatively insensitive to instruction but was predictive of participants’ behaviour on the next trial. The latter finding perhaps accounts for the VALENCE effect in the first analysis: P3 amplitude may be larger for trials with negative than positive feedback because negative trials are more often followed by a reversal in behaviour.

##### 3.1.2.4 P1 and N1 modulation by volatility instructions

To test whether the established FRN effect was modulated by an instruction effect on low-level attention to feedback stimuli, we measured visual P1 and N1 potentials evoked by feedback events. This analysis found no significant effect of INSTRUCTION, or VALENCE, and no interaction between the two on the P1 (all *F*s<1). There was likewise no significant main effect or interaction in the corresponding repeated measures ANOVA for the N1 (all *F*<1). Similar null-effects were established in additional analyses measuring the N1 as base-to-peak amplitude either in this posterior cluster, or in a fronto-central cluster. In sum, the analyses of visual potentials towards feedback events do not suggest that the effects established in the FRN analyses are driven by an attention-orienting effect that differed across instruction conditions.

#### 3.1.3 Experiment 1 summary

Behavioural analysis of Experiment 1 showed that participants integrated instructions and experienced feedback, adapting faster to unannounced rule switches faster under volatility instructions. EEG recordings showed that instructions clearly modulated preparation for stimulus processing, as signified by increased SPN amplitude under volatility instructions. Rapid evaluation of the feedback, reflected in the FRN, showed an integration of experienced feedback and instructions: FRN amplitude was increased under volatility instructions, i.e., when feedback informativeness was increased. P3 amplitude, by comparison, did not vary by instruction, but instead varied as a function of behaviour on the next trial. The lack of difference in visual potentials between instruction conditions, intact learning of the new-mapping following rule reversals in the stability-instructed blocks, and no difference in reaction times between instruction conditions show that these effects are not driven by a lack of task-engagement or attention to the task under stability instruction.

### 3.2 Experiment 2

#### 3.2.1 Experiment 2 – behavioural analysis

The second experiment investigated the effect on feedback processing of instructions about feedback reliability. To create a plausible context for the target instruction conditions, which had identical feedback reliability, we also implemented two conditions that differed with regard to objective feedback reliability. We provide a brief summary of the main comparisons of conditions with objective reliability differences (high reliability vs. low reliability) and then focus on the critical comparisons of blocks with identical objective reliability but different instructions (instructed reliability vs. instructed unreliability), corresponding to the analyses presented for Experiment 1. All statistical analyses, if not stated otherwise, are two-tailed, paired-sample *t*-test, with an alpha-level of 0.05.

##### 3.2.1.1 Performance with different levels of objective feedback reliability

Initial acquisition of the correct mapping showed effects of objective feedback reliability, with significantly higher performance (percent correct) in blocks with reliable (89%, *SEM=*1%) than unreliable feedback (75%, *SEM*=3%; *t*(14)=5.83, *p*<0.01), and fewer initial trials-to-repetition of the correct rule, (2.21, vs. 4.71, trials, *t*(14)=5.51, *p*<0.01). Unreliable feedback also made it harder to adapt behaviour to unannounced changes in task rules, as evident from higher accuracy after rules had reversed in the reliable (85%, *SEM*=1%) than the unreliable feedback blocks (58%, *SEM*=3%; *t*(14)=7.99, *p*<0.01), and fewer trials-to-repetition in reliable (3.62, *SEM*=0.17) compared to unreliable blocks (6.7, *SEM*=0.58; *t*(14)=5.34, *p*<0.01). Lastly, the propensity to switch to an alternative mapping following negative feedback was higher under reliability (20%, *SEM*=2%) than unreliability conditions (14%, *SEM*=3%), although the difference was only marginally significant (*t*(14)=2, *p*<0.1).

##### 3.2.1.2 Experiment 2 – effect of reliability instructions on initial acquisition

Comparing performance in blocks with objectively identical feedback reliability but differing instructions, we found no reliable difference in accuracy between reliability-instruction blocks (86%, *SEM*=1%) than unreliability-instructed blocks (80%, *SEM*=4%; *t*(14)=1.28, *p*=0.22). As hypothesised, and similar to the results of Experiment 1, instructions had no reliable effect on the number of trials to establish the initially correct mapping under instructed reliability (2.7, *SEM*=0.15) than instructed unreliability (3.8, *SEM*=0.69; *t*(14)=1.44, *p*=0.17) (Fig. 2). Finally, instruction effects were evident as the propensity to switch to an alternative mapping following negative feedback was significantly higher (*t*(14)=2.14, *p*<0.05) under reliability instructions (16%, *SEM*=2%) than unreliability instructions (12%, *SEM*=2%).

##### 3.2.1.3 Experiment 2 – effect of instructions on adaptation of behaviour

Participants showed less sensitivity to rule reversals in unreliability-instructed blocks than reliability-instructed blocks. Overall accuracy was numerically higher post-reversal in reliability-instructed blocks than in unreliability-instructed blocks (74% vs. 67%), although this difference did not reach significance (*t*(14)=1.6, *p*=0.26). Reduction in trials-to-repetition of the correct rule reached marginal significance (*t*(14)=1.98, *p*=0.066), with fewer trials in reliability-instructed (4.9, *SEM*=0.43) compared to unreliability-instructed (6.08, *SEM*=0.6) blocks (Fig. 2).

Comparison of adaptation rate measured as trials-to-repetition in the first block and last block of each instruction condition led to slightly less conclusive results than in Experiment 1. There was no significant effect of instruction comparing only the first block of each instruction type in which there was a rule reversal (*t*(14)=0.9, *p*=0.19, Cohen's *d*=0.26). The effect was significant in the last block, however (*t*(14)=2.9, *p*=0.058, Cohen's *d*=0.88). As in Experiment 1, there was no effect of block between the differences found under different instructions (*t*(14)=−1.1, *p*=0.31, Cohen's *d*=−0.37). Again, we thus find no conclusive evidence to suggest that the modulation of behaviour by instructions was altered by long-term experience with the instructions. We note that the power of this statistical test may be limited, as it is based on observations from a single block per condition across 15 participants.

Finally, there were no effects of instruction on the likelihood of participants to reverse their mapping following surprising negative feedback once they had established the new rule (*t*<1); again this effect can be explained by participants understanding that rules would reverse only once during a block.

##### 3.2.1.4 Experiment 2 – no differences in model-free negative RPEs

The same instruction-blind, model-free RL algorithm that was used for Experiment 1 was applied to the data from Experiment 2, and yielded again no difference in average negative RPE amplitude between instruction conditions in trials preceding rule reversals (*t*(14)=1.51, *p*=0.151). Low-level unexpectedness is therefore unlikely to account for any differences in amplitude of relevant EEG components across instruction conditions, as established below.

#### 3.2.2 Experiment 2 – EEG

The EEG analysis in Experiment 2 proceeded in three steps. We first established the effects of differences in objective reliability on the FRN, comparing only the highly reliable and highly unreliable conditions in a 2×2 repeated-measures ANOVA with the factors VALENCE and CONDITION. After establishing the effects of real differences in reliability, we then tested whether instructed reliability would lead to comparable effects on the FRN as instructions on volatility. Third, we again tested whether an effect of directed attention could account for changes in FRN amplitude (measuring N1 and P1) and assessed the pre-reversal effects on P3 amplitude, as in Experiment 1.

##### 3.2.2.1 FRN modulation by objective feedback reliability

Testing for the effects of objective reliability, we found that CONDITION had no significant effect on the size of the FRN (*F*_(1,14)_=2.52, *p*=0.13). Feedback VALENCE had the expected significant effect on the FRN (*F*_(1,14)_=195.39 *p*<0.01), with greater amplitude following negative than positive feedback. Moreover, there was a significant interaction between the two factors (*F*_(1,14)_=13.46, *p*<0.01), indicating that the difference in FRN amplitude between positive and negative feedback was larger when feedback was highly reliable than when it was unreliable.

##### 3.2.2.2 FRN modulation by instructed reliability

**Fig. 6:**
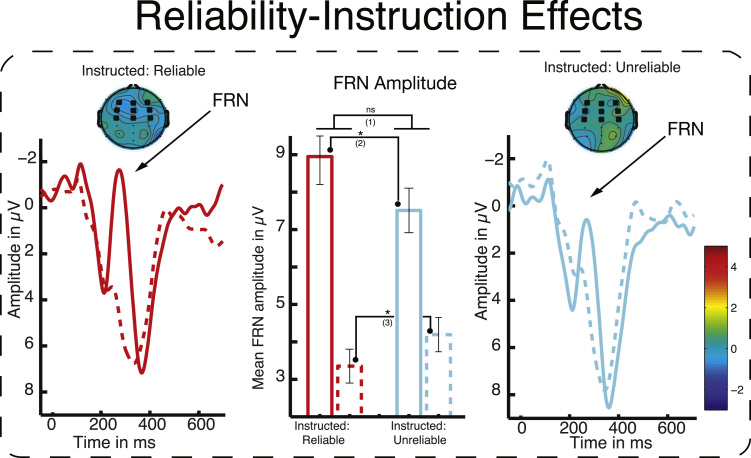
Modulation of the FRN by Reliability Instruction. Time–voltage plots showing the FRN component following positive (dashed lines) and negative (solid lines) unexpected feedback under reliability (left panel) and unreliability (right panel) instructions in the intermediate conditions, which are matched for actual feedback reliability. The bar graphs (middle panel) plot the average over individual amplitudes, showing that there is no significant main effect of instruction on amplitude (1), instead we find the significant interaction between valence and instruction. This interaction is driven by significant difference between FRN amplitude following unexpected negative events in the comparison of reliability-instructed and unreliability-instructed blocks (2), as well as a significant (positive) difference between FRN amplitude following positive feedback under unreliability instruction compared with unexpected negative feedback under reliability instruction. Voltage topographies show the difference between positive and (unexpected) negative feedback under the respective instruction conditions in the time interval between 200 ms and 310 ms post stimulus onset. Dark electrodes delineate clusters that entered the respective statistical analysis and correspond to the electrodes averaged in time–voltage plots. All error bars display standard-error of the mean.

The crucial test for the modulation of the FRN by instructions in Experiment 2, yielded no significant main effect of INSTRUCTION (*F*_(1,14)_=1.2, *p*=0.29), a significant effect of VALENCE (*F*_(1,14)_=82.98, *p*<0.001) and a significant interaction between the two factors (*F*_(1,14)_=9.09 *p*<0.01). A paired *t*-test showed that the difference between instruction conditions was highly significant for negative feedback (*t*(14)=2.38, *p*=0.03; two-tailed), with reliability instructions leading to larger FRN amplitude than unreliability instructions, as predicted. Interestingly, the paired *t*-test for positive feedback showed that the interaction was also influenced by the positive feedback events, which yielded a significant difference in the opposite direction. That is, positive feedback led to a larger FRN under unreliability instructions than under reliability instructions (*t*(14)=−3.21, *p*=.006) (Fig. 6).

##### 3.2.2.3 P3 modulation reflecting behavioural adaptation

As in Experiment 1, overall P3 amplitude following negative and positive feedback was not reliably influenced by instruction: A repeated measures ANOVA with the factors INSTRUCTION and VALENCE yielded no significant effect of INSTRUCTION (*F*_(1,14)_=1.96, *p*=0.18) and contrary to Experiment 1, no effect of VALENCE (*F*<1), and likewise no interaction (*F*<1). As in Experiment 1, we additionally investigated the relationship between P3 amplitude and behavioural adaptation following negative feedback. Here we once again replicated the effect of NEXT TRIAL BEHAVIOUR on P3 amplitude (*F*_(1,14)_=8.75, *p*=0.01), with larger P3 amplitude preceding switches than repetitions of the mapping applied. There was no reliable main effect of INSTRUCTION (*F*<1), but a significant interaction between NEXT TRIAL BEHAVIOUR and INSTRUCTION (*F*_(1,14)_=11.09, *p*<0.01). This interaction indicated that the reversal-related increase in P3 amplitude was greater under reliability-instruction than unreliability-instruction (Fig. 5).

##### 3.2.2.4 P1 and N1 modulation by instructions

Analysis of the P1 and N1 components provided some evidence of differences in low-level attention to feedback as a function of instruction condition. For the P1, we found no significant effect of INSTRUCTION (*F*<1), a significant effect of VALENCE (*F*_(1,14)_=8.074, *p*=0.013), with positive feedback leading to a larger P1 than negative feedback, and a trend-level interaction (*F*_(1,14)_=4.05, *p*=0.063). The interaction was driven by a larger P1 amplitude after positive than negative feedback especially in blocks with reliability instruction compared to blocks with unreliability instruction. For the N1 component, we observed a reliable main effect of VALENCE (*F*_(1,14)_=7.99, *p*=0.013), a main effect of INSTRUCTION (*F*_(1,14)_=7.4, *p*=0.016) and a significant interaction (*F*_(1,14)_=47.14, *p*<0.001). The interaction was driven by a larger N1 following negative feedback than positive feedback, specifically under instructed reliability. Thus, overall in this experiment, it seems that more attention was directed towards feedback events that were expected to be reliable (and which subsequently elicited an enhanced FRN).

#### 3.2.3 Experiment 2 summary

Behavioural analysis of Experiment 2 replicated and extended the major findings of Experiment 1. Instructions that increased the informativeness of the feedback (here, reliability instructions) led to faster adaptation following rule reversals. Further, Experiment 2 replicated the key finding that feedback processing can be modulated by higher-order representations, again showing an increase in FRN amplitude for instructions emphasizing informativeness of the feedback. In contrast to the results of Experiment 1, this FRN modulation was accompanied by reliable changes in early visual potentials evoked by feedback presentation, suggesting differences in the level of attention paid to feedback across instruction conditions. However, behavioural markers (e.g., how quickly the initial mapping is acquired in both conditions) suggest that overall task engagement did not differ as a function of instructed reliability. Finally, this experiment replicated the finding that P3 amplitude was predictive of changes in behaviour on the next trial but, in contrast to Experiment 1, that this effect was modulated by instruction (as a function of the informative value of the feedback).

## 4 Discussion

The present experiments demonstrate consistent influence of high-level belief, manipulated via explicit instruction, on behavioural and neural markers of adaptive learning. Specifically, we assessed the impact of manipulating perceived informative value of trial-by-trial feedback in a novel reversal-learning task, by providing instructions about the volatility of the environment and the reliability of the feedback. We predicted that increased informativeness would change how readily participants adapt behaviour following unexpected feedback, and would modulate processing in a neural system so far predominantly associated with experience-driven reward prediction errors. Both experiments confirmed these predictions, showing that learning is faster and FRN amplitude increases when negative feedback is perceived to be more informative of changes in the environment. These instruction effects were observed in the very first blocks of the experiment, demonstrating that they did not depend on global expectancies built up through participants’ experience with task contingencies, but rather reflected rapid and flexible assimilation of instructed information into the learning process. These changes in learning as a function of perceived informativeness of feedback were reflected in increased amplitude of the FRN component. At the same time, we observed increased preparation for feedback processing as its informational value increased, as reflected in enhanced pre-feedback EEG activity. Together, these findings are indicative of a flexible learning system that integrates instruction and experience to guide adaptive behaviour.

A core component of adaptive behaviour is determining whether unexpected outcomes are a consequence of lasting changes in our environment, or rather reflect chance occurrence. Whereas environmental changes require adaptation, perseverance is crucial in producing effective goal-directed behaviour when faced with random aberrations. High-level knowledge about the informativeness of feedback in a given environment can assist in accurately interpreting that feedback. A key feature of our experimental designs was therefore de-confounding experience-based expectancies and informative value. In Experiment 1, instruction that rules are likely to reverse (high volatility) made negative feedback more informative compared to negative feedback under stability instructions; however, if anything negative feedback was also less surprising under volatility instructions compared to stability instructions. In Experiment 2, instructions indicating increased feedback reliability render negative feedback more surprising and more informative than it appears under unreliability instructions. Both experiments showed that the FRN increased with the informative value of negative feedback, even in the absence of accompanying differences in the expectedness negative feedback (as reflected in overall probability, and in negative reward prediction error derived from a simple model-free reinforcement learning algorithm).

Our findings thus represent a departure from existing characterisations of the FRN-indexed learning system as reflecting a rapid evaluation of experience, with regard to the valence of feedback (Nieuwenhuis et al., 2004, Yeung and Sanfey, 2004) or reward prediction error (Holroyd and Coles, 2002, Walsh and Anderson, 2012; Hauser, 2014 Sambrook and Goslin, 2014). Instead, they suggest that the neural system generating prediction errors is cognitively penetrable and integrates higher-order information in prediction error processing. This conclusion suggests a direct and facilitatory effect of instruction on reinforcement learning, which points to a nuanced picture of the relationship between instruction-based and experience-based learning (cf. O’Reilly, 2013).

On the one hand, previous results seem to suggest independence of model-based processing, which refers to knowledge about the contingencies between events, and model-free processing of experienced feedback. This work proposed a two-stage model of adaptive learning and goal-directed action (Daw et al., 2005, Walsh and Anderson, 2011). Within this framework, responses that are implemented based on instructions (i.e., based on a model of events) override, rather than directly modulate, the computations of model-free reinforcement learning. This account has been supported by evidence that information about the value of choosing a particular stimulus influences choice behaviour but does not modulate FRN amplitude (Walsh and Anderson, 2011). On the other hand, some recent work suggests an antagonistic relationship between model-free and model-based learning, with neural signatures of model-free prediction errors diminished when participants made choices driven by model-based evaluation of stimulus outcomes (Doll et al., 2015). Thus, across different studies, there is evidence that instruction and experience work in concert (as in the present experiments), that they can operate largely independently (Walsh and Anderson, 2011), or that they are mutually inhibitory (Doll et al., 2015).

We interpret these findings and theories as consistent rather than contradictory, specifically by pointing to the flexibility of the learning process according to current task demands: When instructions are valid and render feedback irrelevant to choice, optimal behaviour relies on implementing the instruction and essentially ignoring the feedback, so integration of experience and instruction and not required (Walsh and Anderson, 2011). Conversely, when model-based evaluation and model-free learning are equally suited to solve a task, it seems that the model-based system will inform the model-free learner to the degree to which the higher-order system is involved in selecting actions (Doll et al. 2015). This finding of possible communication between systems is consistent with our results. However, our paradigm is unique in that optimal behaviour relies on integration of information from two different sources—participants use a model of the world (based on instructions) to inform their interpretation of experienced low-level contingencies (based on feedback), rather than trading-off the utility of information from high-level representations and low-level contingencies. This conclusion considerably extends existing knowledge in showing that higher-order representations can amplify, rather than diminish prediction error processing.

An interesting tangent in this regard is work that characterises prediction errors as markers of the salience of external events, rather than as indices of the valence of feedback (Redgrave and Gurney, 2006). In the context of this idea, our findings would imply that informativeness is a high-level source of salience, which constitutes an unsigned, valence-unrelated quality modulating the neural response to feedback above and beyond the effects of low-level unexpectedness (unsigned surprise).

The neural mechanisms underlying integration of instruction-modulated and experience-driven learning is likely to involve a functional interplay between the prefrontal cortex and the basal ganglia. The basal ganglia are classically associated with model-free prediction errors; while the FRN is understood to be generated in the anterior cingulate cortex (Hauser et al., 2014), it is assumed to relate to the output of basal ganglia computations (Foti et al., 2011, Hauser et al., 2014, Holroyd and Coles, 2002). We thus add to recent work, as our results suggest that basal ganglia processing is informed by high-level beliefs from instruction; previous work has suggested that these high-level representation likely depend on flexible representations in prefrontal cortex (Doll, 2011; Stocco et al., 2010, Stocco et al., 2012; Chatham et al., 2014; Mestres-Misse et al., 2016). If this is the case, one mechanism by which modulation could be achieved is through PFC influence on striatal processing as observed by Li et al. (2011).

Further work that supports the link between basal-ganglia prediction errors and higher-order beliefs comes from a recent combination of computational modelling and genotyping: Participants of a genotype that diminishes the striatal response to unexpected negative events find it harder to re-learn the actual worth of a stimulus after receiving false information (Doll et al., 2011). Further, patients with schizophrenia, a neurological condition associated with a change in dopaminergic innervation of the prefrontal cortex (Doll et al., 2014), are less susceptible to (false) instructed beliefs about the value of a stimulus than healthy controls. Together, these results suggest interplay of basal ganglia and prefrontal computations where, on the one hand, prefrontal modulation provides an additional input to basal ganglia computations. On the other hand, tracking of prediction errors in the basal ganglia can reverse the influence of false higher-order information (Doll et al., 2011). Our results go further in providing evidence that prediction error signals, which constitute the output of the basal ganglia, are informed by prefrontal input when integration of experience and higher-order knowledge is essential for optimal behaviour in the task. In this context, however, we note that the relationship between basal ganglia prediction errors and the FRN remains a topic of debate, and information transfer between these network components may be bi-directional (Frank et al., 2005, Cavanagh and Frank, 2014). Whether integration of higher-order and low-level information is achieved at the stage of the basal ganglia computation, or within the PFC, is a key question for future work.

Regardless, the mechanistic implication of this model is that the integrated learning system is proactive in selecting relevant information to guide learning. We find evidence of this active preparation for processing learning-relevant feedback in modulations of the SPN component (Kotani et al., 2013), which we have shown to be influenced by current beliefs regarding the informative value of feedback. This effect was observed in the absence of consistent modulation of early visual potentials, suggesting that preparation does not simply entail low-level attentional adjustments. Rather, we find a modulation preceding the sampling process by interpretation of the anticipated relevance of feedback for adaptive behaviour.

The suggestion that integration of higher-order beliefs modulates behaviour is consistent with findings from our Hidden Markov Model (HMM) comparison. Here, we modelled the impact of volatility instructions as increasing the learner's aversion towards uncertainty caused by unexpected feedback. An implication of this approach is that instructions modulate how experience is interpreted to form action policies, rather than modulating state estimations (e.g., of the likelihood of negative vs. positive feedback). Indeed, we found that the FRN amplitude did not predict behaviour on the next trial, suggesting that although this signal integrates higher-order beliefs and experience, the behavioural effect of instructions may be driven by a modulation of a parameter at a later stage in the action selection hierarchy. However, it remains for future work to test formally whether artificial learners that focus on the integration-stage could predict behaviour better than learners in which instruction alters parameters of action selection, and whether neural markers of the selection stage vary according to beliefs.

Both of the present experiments replicated the finding that P3 amplitude following negative feedback increases when participants’ choose to change strategy on the following trial (Chase et al., 2011). As previously mentioned, no close link to trial-by-trial behaviour was apparent in the FRN. We interpret this finding within the framework of the P3 as a marker of decision-making which holds that P3 amplitude reflects the accumulation of evidence in favour of one decision (e.g., stay or switch) over another (O'Connell et al., 2012). The nature of the study does not allow us to discriminate whether the P3 amplitude reflects behavioural adaptation as a global process, or is limited to rule-switching.

Contrary to the FRN, this P3 effect did not consistently vary according to participants’ beliefs about the informativeness of the current feedback: We found modulation of P3 amplitude only with instructions about feedback reliability, and not environment volatility. A possible explanation for this difference is that if the P3 in fact tracks evidence for the correctness of a foregoing decision, this tracking may be influenced by information about the evidence itself (i.e., the feedback reliability), but not to the same degree by information about the environment in which this evidence occurs (i.e., information in volatility of the environment).

## Conclusion

We used instructions about the environment as a canonical form of high-level influence in a task requiring flexible adaptation of behaviour. Our experiments show that instructions about higher-level features of the environment can change neural processing of action outcomes. In light of the present findings, and against the backdrop of previous work, we argue that experience of outcomes and instruction can mutually inform each other to promote flexible, adaptive behaviour. Clearly, instructions are just one, arguably uniquely human, source of higher-order representation. Past experience can likewise aggregate to higher-order representations, shaping expectations that can in turn modulate how the surprise associated with immediate feedback is interpreted.

Collectively, these computations solve the task of determining the significance of unexpected events. This flexibility allows human learners to successfully navigate in our complex, volatile environments, and to make informed decisions about whether to persevere or explore new options when we are surprised by the consequences of our actions. Future work will need to address the neural basis of this flexible learning, testing whether informativeness-modulated surprise signals are generated within the prefrontal-basal ganglia network as we propose above, and whether neural correlates of action selection reflect parameters that predict behaviour. Combining computational models of behaviour with trial-by-trial measures of neural variability, such as afforded by fMRI and MEG, appears the most promising approach to uncover the foundations underlying this type of flexible behaviour.

## Conflict of interest

The authors declare no competing financial interests.

## References

[bib2] Behrens T.E.J., Woolrich M.W., Walton M.E., Rushworth M.F.S. (2007). Learning the value of information in an uncertain world. Nat. Neurosci..

[bib3] Bland A.R., Schaefer A. (2011). Electrophysiological correlates of decision making under varying levels of uncertainty. Brain Res..

[bib4] Botvinick M., Weinstein A. (2014). Model-based hierarchical reinforcement learning and human action control. Philos. Trans. R. Soc. B.

[bib5] Brainard D.H. (1997). The psychophysics toolbox. Spat. Vis..

[bib6] Brunia C.H.M. (1988). Movement and stimulus preceding negativity. Biol. Psychol..

[bib7] Cavanagh J.F., Frank M.J. (2014). Frontal theta as a mechanism for cognitive control. Trends Cognit. Sci..

[bib8] Chase H.W., Swainson R., Durham L., Benham L., Cools R. (2011). Feedback-related negativity codes prediction error but not behavioral adjustment during probabilistic reversal learning. J. Cognit. Neurosci..

[bib9] Chatham C.H., Frank M.J., Badre D. (2014). Corticostriatal output gating during selection from working memory. Neuron.

[bib10] Cole M.W., Laurent P., Stocco A. (2013). Rapid instructed task learning: a new window into the human brain's unique capacity for flexible cognitive control. Cognit. Affect. Behav. Neurosci..

[bib11] Daw N.D., Niv Y., Dayan P. (2005). Uncertainty-based competition between prefrontal and dorsolateral striatal systems for behavioral control. Nat. Neurosci..

[bib12] Delorme A., Makeig S. (2004). EEGLAB: an open source toolbox for analysis of single-trial EEG dynamics including independent component analysis. J. Neurosci. Methods.

[bib13] Doll B.B., Duncan K.D., Simon D.A., Shohamy D., Daw N.D. (2015). Model-based choices involve prospective neural activity. Nat. Neurosci..

[bib14] Doll B.B., Hutchison K.E., Frank M.J. (2011). Dopaminergic genes predict individual differences in susceptibility to confirmation bias. J. Neurosci..

[bib15] Doll B.B., Waltz J.A., Cockburn J., Brown J.K., Frank M.J., Gold J.M. (2014). Reduced susceptibility to confirmation bias in schizophrenia. Cognit. Affect. Behav. Neurosci..

[bib16] Eimer, M., 2014. In: K. Nobre S. Kastner(Eds.), The Time Course of Spatial Attention: Insights from Event-related Brain Potentials. Oxford Handbook of Attention.

[bib17] Foti D., Weinberg A., Dien J., Hajcak G. (2011). Event-related potential activity in the basal ganglia differentiates rewards from nonrewards: temporospatial principal components analysis and source localization of the feedback negativity. Hum. Brain Mapp..

[bib18] Gehring W.J., Willoughby A.R. (2002). The medial frontal cortex and the rapid processing of monetary gains and losses. Science.

[bib19] Ghahramani Z. (2001). An introduction to hidden Markov models and Bayesian networks. Int. J. Pattern Recognit. Artif. Intell..

[bib20] Hampton A.N., Bossaerts P., O’doherty J.P. (2006). The role of the ventromedial prefrontal cortex in abstract state-based inference during decision making in humans. J. Neurosci..

[bib21] Hauser T.U., Iannaccone R., Stämpfli P., Drechsler R., Brandeis D., Walitza S., Brem S. (2014). The feedback-related negativity (FRN) revisited: new insights into the localization, meaning and network organization. Neuroimage.

[bib22] Holroyd C.B., Coles M.G.H. (2002). The neural basis of human error processing: Reinforcement learning, dopamine, and the error-related negativity. Psychol. Rev..

[bib23] Kass R.E., Raftery A.E. (1995). Bayes factors. J. Am. Stat. Assoc..

[bib24] Kotani Y. (2003). Effects of information and reward on stimulus-preceding negativity prior to feedback stimuli. Psychophysiology.

[bib25] Li J., Delgado M.R., Phelps E.A. (2011). How instructed knowledge modulates the neural systems of reward learning. Proc. Natl. Acad. Sci. USA.

[bib26] Luck S.J., Woodman G.F., Vogel E.K. (2000). Event-related potential studies of attention. Trends Cognit. Sci..

[1] Mestres-Missé, A., Trampel, R., Turner, R., Kotz, S.A., 2016. Uncertainty and expectancy deviations require cortico-subcortical cooperation. NeuroImage. 10.1016/j.neuroimage.2016.05.069 27261161

[bib27] Miltner W.H., Braun C.H., Coles M.G. (1997). Event-related brain potentials following incorrect feedback in a time-estimation task: Evidence for a “generic” neural system for error detection. J. Cognit. Neurosci..

[bib28] Morís J., Luque D., Rodríguez-Fornells A. (2013). Learning-induced modulations of the stimulus-preceding negativity. Psychophysiology.

[bib29] Näätänen R., Picton T.W. (1987). The N1 wave of the human electric and magnetic response to sound: a review and an analysis of the component structure. Psychophysiology.

[bib30] Nieuwenhuis S., Yeung N., Holroyd C.B., Schurger A., Cohen J.D. (2004). Sensitivity of electrophysiological activity from medial frontal cortex to utilitarian and performance feedback. Cereb. Cortex.

[bib31] O'Connell R.G., Dockree P.M., Kelly S.P. (2012). A supramodal accumulation-to-bound signal that determines perceptual decisions in humans. Nat. Neurosci..

[bib32] O’Reilly J.X. (2013). Making predictions in a changing world – inference, uncertainty, and learning. Front. Neurosci..

[bib34] Polich J. (2007). Updating P300: an integrative theory of P3a and P3b. Clin. Neurophysiol..

[bib35] Redgrave P., Gurney K. (2006). The short-latency dopamine signal: a role in discovering novel actions?. Nat Rev. Neurosci..

[bib36] Sambrook T.D., Goslin J. (2014). A neural reward prediction error revealed by a meta-analysis of ERPs using great grand averages. Psychol. Bull..

[bib37] Schultz W., Dayan P., Montague P.R. (1997). A neural substrate of prediction and reward. Science.

[bib39] Semlitsch H.V., Anderer P., Schuster P., Presslich O. (1986). A solution for reliable and valid reduction of ocular artifacts, applied to the P300 ERP. Psychophysiology.

[1] Shannon, C.E., 1948. A Mathematical Theory of Communication, Bell System Technical Journal 27, 379–423 & 623–656.

[bib40] Sutton R.S., Barto A.G., Gabriel M., Moore J. (1990). Time-derivative models of Pavlovian reinforcement. Learning and Computational Neuroscience: Foundations of Adaptive Networks.

[bib41] Stocco A., Lebiere C., O’Reilly R.C., Anderson J.R. (2012). Distinct contributions of the caudate nucleus, rostral prefrontal cortex, and parietal cortex to the execution of instructed tasks. Cognit. Affect. Behav. Neurosci..

[bib42] Stocco A., Lebiere C., Anderson J.R. (2010). Conditional routing of information to the cortex: A model of the basal ganglia's role in cognitive coordination. Psychol. Rev..

[bib43] Walsh M.M., Anderson J.R. (2011). Modulation of the feedback-related negativity by instruction and experience. Proc. Natl. Acad. Sci. USA.

[bib44] Walsh M.M., Anderson J.R. (2012). Learning from experience: event-related potential correlates of reward processing, neural adaptation, and behavioral choice. Neurosci. Biobehav. Rev..

[bib45] Widmann A., Schröger E. (2012). Filter effects and filter artifacts in the analysis of electrophysiological data. Front. Psychol..

[bib46] Yeung N., Sanfey A.G. (2004). Independent coding of reward magnitude and valence in the human brain. J. Neurosci..

[1] Yu, A., Dayan, P., 2003. Expected and unexpected uncertainty: ACh and NE in the neocortex. Advances in neural information processing systems, 173–180.

