# Supplementary material for: Adaptive behaviour and feedback processing integrate experience and instruction in reinforcement learning
Source: Neuroimage. 2017 Feb 1;146:626–41. doi: 10.1016/j.neuroimage.2016.08.057 (PMC5312784; doi:10.1016/j.neuroimage.2016.08.057)
Supplement: Supplementary file 1 — Supplementary material [file mmc1.docx]

**Supplemental**

**Model description**

**We tested whether an artificial learner sensitive to instructions describes behaviour in the task better than an agent with an instruction blind algorithm; to this end we compared two Bayesian Hidden State Markov Models (HMM; Gharamani, 2001; Hampton et al., 2006). These models differed with regard to whether they were instruction blind (basis model), or instruction sensitive (instruction model).**

**In the formal account, X(t) represents the two abstract, hidden states (i,j) at trial t; in state i, mapping 1 (making a right-hand response to stimulus A and left-hand response to stimulus B) is the correct underlying mapping driving the feedback towards participants responses. In state j, mapping 2 is the correct underlying mapping driving the feedback (requiring left-hand responses to stimulus B and right-hand responses to stimulus A – the participants are naturally unaware which stimulus is stimulus A, or B). In close keeping with the Hampton et al. model, Y(t) represent the feedback (positive or negative) participant receive at time t. S(t) represent whether they’ve just switched the mapping or stayed with the mapping they implemented on the last trial. The conditional probabilities linking these variables are as follows:**

$$\boldsymbol{P}\left( \boldsymbol{X}_{\boldsymbol{t}} | \boldsymbol{X}_{\boldsymbol{t-1}}\boldsymbol{,}\boldsymbol{S}_{\boldsymbol{t}}\boldsymbol{=stay} \right)\boldsymbol{=}\left( \begin{matrix} \boldsymbol{1-\partial} & \boldsymbol{\partial} \\ \boldsymbol{\partial} & \boldsymbol{1-\partial} \end{matrix} \right)\boldsymbol{,}$$

$$\boldsymbol{P}\left( \boldsymbol{X}_{\boldsymbol{t}} | \boldsymbol{X}_{\boldsymbol{t-1}}\boldsymbol{,}\boldsymbol{S}_{\boldsymbol{t}}\boldsymbol{=switch} \right)\boldsymbol{=}\left( \begin{matrix} \boldsymbol{\partial} & \boldsymbol{1-\partial} \\ \boldsymbol{1-\partial} & \boldsymbol{\partial} \end{matrix} \right)\boldsymbol{,}$$

**(1)**

**∂ is the reversal probability, was fixed at the frequentist probability of any trial being a switch trial, given by the proportion of all trials which were switch trials. The probability that feedback is indicative of the true state of the world is given by its reliability, P(Y_t_|X_t_ = correct) = 0.75.**

**As in Hampton et al., each block started with a prior of P(X_0_) = (0.5,0.5). Bayesian inference was performed on a trial-by-trial basis to calculate the posterior probability of the random variable given the feedback and the subjects’ behaviour on the last trial (variable S); this inference uses causal belief propagation (Eqs. 2,3) in a non-leaky model.**

**The posterior is calculated as**

$$\boldsymbol{Posterior}\left( \boldsymbol{X}_{\boldsymbol{t}}\boldsymbol{=correct} \right)\boldsymbol{=}\frac{\boldsymbol{P(}\boldsymbol{Y}_{\boldsymbol{t}}\boldsymbol{|}\boldsymbol{X}_{\boldsymbol{t}}\boldsymbol{=correct)Prior(}\boldsymbol{X}_{\boldsymbol{t}}\boldsymbol{=correct)}}{\sum_{\boldsymbol{X}_{\boldsymbol{t}}\boldsymbol{states}} \boldsymbol{P(}\boldsymbol{X}_{\boldsymbol{t}}\boldsymbol{|}\boldsymbol{Y}_{\boldsymbol{t}}\boldsymbol{)Prior(}\boldsymbol{X}_{\boldsymbol{t}}\boldsymbol{=correct)}}$$

**, (2)**

**and this posterior value is then updated by the switch probability and entered as a prior in the next trial.**

$\boldsymbol{Prior}\left( \boldsymbol{X}_{\boldsymbol{t}}\boldsymbol{=correct} \right)\boldsymbol{=}\sum_{\boldsymbol{X}_{\boldsymbol{t-1 state}}} \boldsymbol{Posterior(}\boldsymbol{X}_{\boldsymbol{t-1}}\mathbf{)}\boldsymbol{P}\left( \boldsymbol{X}_{\boldsymbol{t}}\boldsymbol{=correct} \right| \boldsymbol{X}_{\boldsymbol{t-1}}\boldsymbol{,}\boldsymbol{S}_{\boldsymbol{t}}\boldsymbol{)}$ **, (3)**

**The models, base their decision to switch to a new mapping on an individually fitted level of aversion, α, towards Entropy, H. Entropy is a measure of the increase (or decrease) of uncertainty produced by the accumulation of information over trials (Shannon, 1949). Information, *I*, associated with each outcome is given by**

$\boldsymbol{I}_{\boldsymbol{t}}\boldsymbol{= -}\mathbf{log}\boldsymbol{(Posterior}\left( \boldsymbol{X}_{\boldsymbol{t}}\boldsymbol{=correct} \right)\boldsymbol{)}$ **, (4)**

**The terminology of *information*, or *surprise* for this quantity follows information theory (Shannon, 1949); please note that this quantity is different from the concept of informativeness, and different from low-level unexpectedness implemented in this study.**

**Entropy is then measured as**

$\boldsymbol{H}_{\boldsymbol{t}}\boldsymbol{=-}\sum_{\boldsymbol{i}}^{\boldsymbol{states}} \mathbf{log}\boldsymbol{(Posterior}\left( \boldsymbol{X}_{\boldsymbol{t}}\boldsymbol{=correct} \right)\boldsymbol{)(Posterior}\left( \boldsymbol{X}_{\boldsymbol{t}}\boldsymbol{=correct} \right)$**, (5)**

**An illustration of the relationship of response mappings, feedback, information, and entropy is provided in Figure 3 a.**

**The decision rule is given by the softmax function**

$\boldsymbol{P}\left( \boldsymbol{switch}_{\boldsymbol{t}} \right)\boldsymbol{=}\frac{\mathbf{}\boldsymbol{e}^{\boldsymbol{\beta(}\boldsymbol{H}_{\boldsymbol{t}}\boldsymbol{- \alpha)}}}{\boldsymbol{e}^{\boldsymbol{\beta(}\boldsymbol{H}_{\boldsymbol{t}}\boldsymbol{- \alpha)}}\boldsymbol{+}\boldsymbol{e}^{\boldsymbol{\beta(1-}\left( \boldsymbol{H}_{\boldsymbol{t}}\boldsymbol{- \alpha} \right)\boldsymbol{)}}}\boldsymbol{,}$**(6).**

**Simulated annealing was used for model fitting. The instruction-blind model fitted parameters α and β individually to maximise the percentage of correctly predicted responses for each participant in each block, regardless of the type of block or instruction. The instruction sensitive model implemented the individually fitted β parameters from the instruction-blind model and fitted two parameters, α_s_ (under stability instruction) and α_v_ (under volatility instructions), individually in the according simulated annealing procedure maximising the percentage of correctly predicted responses per block, weighing all blocks equally. However, entropy avoidance parameters α_s_ & α_v_ were allowed, albeit not forced to vary between blocks with volatility instruction and blocks with stability instruction. Note that this fitting explicitly refers to the instruction, not the actual level of volatility in the blocks, closely mirroring participants’ beliefs.**
